# Unraveling Protein Networks with Power Graph Analysis

**DOI:** 10.1371/journal.pcbi.1000108

**Published:** 2008-07-11

**Authors:** Loïc Royer, Matthias Reimann, Bill Andreopoulos, Michael Schroeder

**Affiliations:** Biotechnology Center, Technische Universität Dresden, Germany; University of Cologne, Germany

## Abstract

Networks play a crucial role in computational biology, yet their analysis and representation is still an open problem. Power Graph Analysis is a lossless transformation of biological networks into a compact, less redundant representation, exploiting the abundance of cliques and bicliques as elementary topological motifs. We demonstrate with five examples the advantages of Power Graph Analysis. Investigating protein-protein interaction networks, we show how the catalytic subunits of the casein kinase II complex are distinguishable from the regulatory subunits, how interaction profiles and sequence phylogeny of SH3 domains correlate, and how false positive interactions among high-throughput interactions are spotted. Additionally, we demonstrate the generality of Power Graph Analysis by applying it to two other types of networks. We show how power graphs induce a clustering of both transcription factors and target genes in bipartite transcription networks, and how the erosion of a phosphatase domain in type 22 non-receptor tyrosine phosphatases is detected. We apply Power Graph Analysis to high-throughput protein interaction networks and show that up to 85% (56% on average) of the information is redundant. Experimental networks are more compressible than rewired ones of same degree distribution, indicating that experimental networks are rich in cliques and bicliques. Power Graphs are a novel representation of networks, which reduces network complexity by explicitly representing re-occurring network motifs. Power Graphs compress up to 85% of the edges in protein interaction networks and are applicable to all types of networks such as protein interactions, regulatory networks, or homology networks.

## Author Summary

Networks play a crucial role in biology and are often used as a way to represent experimental results. Yet, their analysis and representation is still an open problem. Recent experimental and computational progress yields networks of increased size and complexity. There are, for example, small- and large-scale interaction networks, regulatory networks, genetic networks, protein-ligand interaction networks, and homology networks analyzed and published regularly. A common way to access the information in a network is though direct visualization, but this fails as it often just results in “fur balls” from which little insight can be gathered. On the other hand, clustering techniques manage to avoid the problems caused by the large number of nodes and even larger number of edges by coarse-graining the networks and thus abstracting details. But these also fail, since, in fact, much of the biology lies in the details. This work presents a novel methodology for analyzing and representing networks. Power Graphs are a lossless representation of networks, which reduces network complexity by explicitly representing re-occurring network motifs. Moreover, power graphs can be clearly visualized: they compress up to 90% of the edges in biological networks and are applicable to all types of networks such as protein interaction, regulatory networks, or homology networks.

## Introduction

In recent years, novel high-throughput methods, such as yeast two-hybrid assays [1] and affinity purification techniques [2],[3], have been used to characterize protein interactions at a large scale and have produced a wealth of data in the form of networks of interacting proteins. Comprehensive protein interaction networks have been assembled for several species: *S. cerevisiae* [4]–[6], *C. elegans* [7], *D. melanogaster* [8],[9], *H. pylori* [10], *H. sapiens* [11],[12], and *P. falciparum* [13]. Networks are also obtained with other high-throughput data collection methods, either experimentally or in silico, such as ChIP-on-chip [14] experiments, whole interactome scanning experiments (WISE) [15], sequence homology networks [16] and others. The challenge remains to obtain biological insights through the analysis of these networks.

In the case of protein interaction networks, their topology has been explored through the clustering of proteins into groups that share the same biological function, are similarly localized in the cell, or are part of a complex. To this end, several algorithms have been developed, such as socio-affinity clustering [4], the Restricted Neighborhood Search Clustering (RNSC) algorithm [17], the MCODE algorithm [18], statistical sub-complexes [19], modular decomposition [20] or the MULIC clustering algorithm [21].

**Figure 1:**
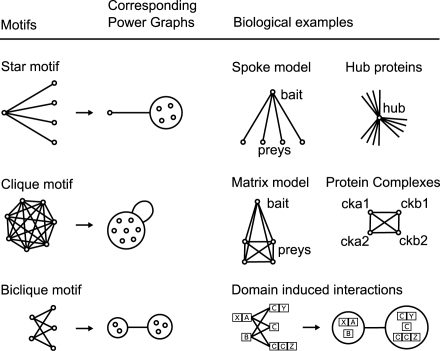
The Three Basic Motifs: Star, Biclique, and Clique. Stars often occur because of hub proteins or when affinity purification complexes are interpreted using the spoke model. Bicliques often arise because of domain-domain or domain-motif interactions inducing protein interactions [25]. Power nodes are sets of nodes and power edges connect power nodes. A power edge between two power nodes signifies that all nodes of the first set are connected to all nodes of the second set. Note that nodes within a power node are not necessarily connected to each other.

How does the underlying biology manifest itself in protein interaction networks? Fig. 1 illustrates three recurrent motifs that have been reported in the literature. The first motif is the star, representing a hub protein, which is frequently present in scale-free biological networks [22]. Evolutionary models based on gene duplication, divergence [23] and preferential attachment [24] can explain the abundance of hub proteins. The second motif is the biclique, also referred to as complete bipartite graph: all proteins in one group interact with all proteins in another group. Domain interactions have been reported to induce the occurrence of bicliques. Models of protein interaction networks based on interacting domains have been proposed in which complementary domains are shown to induce bipartite structures [25],[26]. Similarly, bicliques detected in protein interaction networks were used to discover motif pairs at interaction sites [27]. In general, domain interactions and protein interactions have been shown in many studies to be sufficiently correlated to allow domain bindings to be used to predict protein interactions, and conversely, protein interactions to predict domain interactions [28]–[38]. The third motif is the clique, also referred to as complete graph: a set of completely interconnected proteins. In the core of molecular complexes, where the distinction between direct and indirect physical interactions is often blurred, protein interactions are observed to organize into cliques and bicliques. Indeed, the completion of quasi-cliques and quasi-bicliques has been shown to successfully predict missing interactions between proteins [39]. Cliques are a special case of reflexive bicliques. Similarly, stars are also a special case of bicliques in which one node is connected to several other nodes.

The abundance of stars, cliques, and bicliques suggests that modeling protein interaction networks as a collection of binary interactions is an obstacle toward a detailed analysis of the wealth of information contained in high-throughput networks. These networks have many edges that redundantly diffuse the information instead of highlighting it. In this study we introduce a new network representation and analysis paradigm that not only groups proteins into biologically relevant modules but also conveys in all detail–without loss of information–and with fewer symbols, the subtle connection patterns within and between groups of proteins.

## Results and Discussion

### Power Graph Analysis

*Power Graphs* are novel representations of graphs that rely on two abstractions: *power nodes* and *power edges*. *Power nodes* are sets of nodes brought together and power edges connect two power nodes thus signifying that all nodes contained in the first power node are connected to all the nodes contained in the second power node. These language primitives allow for the succinct representation of stars, bicliques and cliques.

As Fig. 1 shows, a star is expressed as a node connected via a power edge to a power node, a biclique is expressed as two power nodes connected by a power edge, and a clique is a power node connected to itself by a power edge. In Fig. 1, the power graph representation reduces the number of edges needed to represent the network, groups together highly connected nodes as well as nodes having similar neighbours, and this without any loss of information. In the following, we will often use the notion of edge reduction i.e. the proportion of edges that are abstracted from the original network in the power graph representation.

*Power Graph Analysis* is the computation and analysis of power graphs. We propose an algorithm that computes power graphs. Node clustering, module detection, network motif composition, network visualization, and network models can be recast in terms of Power Graph Analysis. In the following we demonstrate how power graphs facilitate the task of uncovering underlying biology.

### Understanding Interactions within Molecular Complexes with Power Graphs

Some recent large-scale experiments [4] specifically aim at identifying complexes instead of binary interactions. Complexes are difficult to interpret from the point of view of binary interactions: are two proteins *p_1_* and *p_2_* participating in a complex *C* but not in direct physical contact, interacting?

This point is crucial for the interpretation of results from pull-down assays where whole complexes are identified rather than binary interactions [2],[3]. In a pull-down assay, a purified and tagged protein, the bait, is used to capture other proteins: the preys. These observed complexes are either modelled as cliques in the *matrix* model, or as stars in the *spoke* model [40]. In the case of the spoke model the bait is at the centre of the star, and the preys are linked to it. In the matrix model, all proteins are linked together, signifying that they belong to the same observed complex.

The problem with this perspective is that the spoke model underestimates, and the matrix model overestimates the number of true physical interactions between the members of a complex. For both models the use of binary interactions does not convey succinctly an otherwise simple connection pattern. Let *n* be the number of proteins in the complex. The matrix model represents the complex with a quadratic number of interacting pairs: *n(n−1)/2*. The spoke model requires only *n−1* interacting pairs to represent the same complex. Fig. 1 shows that the power graph representation mitigates this problem: in both cases only one power edge is needed. In the case of the matrix model all proteins are brought together in one power node, whereas in the spoke model the bait protein is on its own and all preys are together in a power node. Let us consider two examples.

#### Example 1—Casein kinase II complex

**Figure 2:**
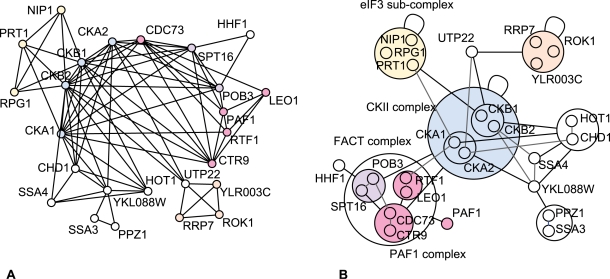
Casein Kinase II Complex. Two catalytic alpha subunits (CKA1, CKA2) and two regulatory beta subunits (CKB1, CKB2) interacting with the FACT complex, with sub-complex NIP1-RPG-PRT1, and with the PAF1 complex. The graph representation (A) consists of 80 edges whereas the power graph representation (B) has 30 power edges, thus an edge reduction of 62%. This simplification of the representation makes the separation of the regulatory subunits from the catalytic subunits immediately apparent without loss of information on individual interactions.

A recent survey of the yeast proteome investigated the modularity of the yeast cell machinery [4]. Fig. 2 shows the casein kinase II complex and its neighbouring complexes. Casein kinase II has been implicated in cell cycle control, DNA repair, regulation of the circadian rhythm and other cellular processes. It is a tetramer of two catalytic alpha subunits CKA1, CKA2 and two regulatory beta subunits CKB1 and CKB2. Remarkably, the power graph representation conveys immediately the difference between the alpha and beta pairs of subunits: the two alpha subunits are grouped together by one power node, and the beta subunits are grouped together by another power node. The reason for this is that the two alpha subunits have almost identical neighbours, which are in turn different from the neighbours shared by the beta subunits. The beta subunits are connected to the eIF3 sub-complex (NIP1, RPG1, PRT1) known to stimulate the binding of mRNA to ribosomes, and through the intermediary protein UTP22 to a power node consisting of proteins ROK1, RRP7 and YLR003C that do not correspond to a known complex but that are all related to RNA processing, possibly a small complex. In contrast, the alpha subunits do not interact with these two groups, but instead with YKL088W an uncharacterized enzyme.

Other complexes are visible in the power graph representation. For example, the proteins POB3 and SPT16 are grouped together in one power node. They form a complex known as the heterodimeric FACT complex SPT16/POB3, a complex involved in the transcription elongation on chromatin templates. It is known that the casein kinase II complex activates the FACT complex [41]. Finally, a group of two power nodes linked by a power edge, all of them interacting with the protein PAF1, form the PAF1 complex–a complex that associates with RNA polymerase II [42].

Overall we see that the power graph representation manages to give an insightful picture of the underlying biology. It should be stressed that these representations are obtained without the addition of biological background knowledge but instead based on the network topology alone. Power Graphs thus provide useful hints into the existence of complexes, their internal organization, and their relationships.

Importantly, the power graph representation is a lossless representation, meaning that all and only interactions from the original network are represented faithfully, which is usually not the case for most clustering methods.

#### Example 2—Untangling the nucleosome

**Figure 3:**
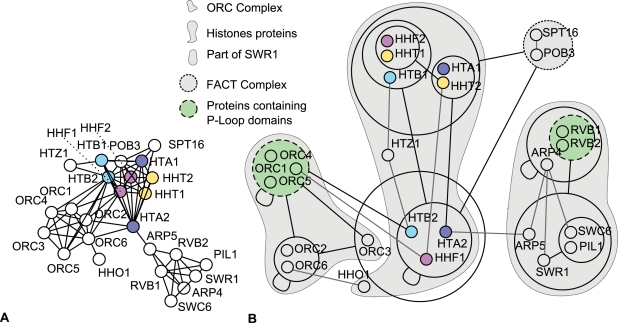
Histone Protein Interactions and Neighbouring Proteins according to Krogan et al. [6]. (A) Standard graph representation. (B) power graph representation. The ORC complex is visible with a power node of proteins–ORC1/ORC4/ORC5–carrying a nucleotide binding P-loop domain [SCOP:52540]. Histones subtypes HTA1/2, HTB1/2, HHT1/2, and HHF1/2 share the same color. Histones HTA2, HTB2 and HHF1 are segregated from their twin subtypes HTA1, HTB1 and HHF2. The FACT complex SPT16/POB3 is again delineated.

Similarly to the survey of the yeast proteome by Gavin et al. [4], Krogan et al. [6] have investigated protein interactions using tandem affinity purification (TAP). Fig. 3A shows a subgraph of proteins surrounding the H1, H2A, H2B, H3 and H4 histone proteins. These proteins form the nucleosome, an octameric complex responsible for the packing of DNA into chromosomes. Interestingly, the subunits H2A, H2B, H3, and H4 come in pairs: HTA1/HTA2 HTB1/HTB2 HHT1/HHT2 and HHF1/HHF2. This is an example of gene duplication [23] inducing a complete bipartite subgraph (biclique) of interactions between proteins expressing duplicated genes. In yeast, HTA1, HTA2, HTB1, and HTB2 are nearly identical, with two and respectively four amino acids differing. HHF1 and HHF2 are identical proteins coded by different genes.

Interacting with histones is the ORC Complex (Origin Recognition Complex) responsible for marking origin regions prior to DNA replication. On Fig. 3B the corresponding power graph is shown. The ORC complex is a clique of six proteins, which appears in the power graph representation as three power nodes linked by three power edges. One of these power nodes–ORC1/ORC4/ORC5–interacts with HTB2 and is enriched in a specific domain: a nucleotide binding P-loop domain containing nucleotide triphosphate hydrolases.

Surprisingly, histones HTA2, HTB2 and HHF1 are segregated from their twin subtypes HTA1, HTB1 and HHF2, as subunits ORC2 and ORC6 interact with HTA2, HTB2 and HHF1 and not with the HTA1, HTB1, and HHF2. This is contradictory to the identity/near identity of these pairs of histones. The power graphs shows the separation between these two types of histones.

Why have these mostly identical proteins different interaction partners? In the case of H2A histones, each subtype has been shown to be sufficient for cell viability, and no clear functional difference were reported apart from homozygous strains for hta1^−^ exhibiting a slower growth [43]. Despite the near identity of these proteins, their interaction profiles are different which suggests that the interactions with ORC2 and ORC6 are false positives or false negatives–all or none of the histones interact with ORC2 and ORC6.

Yet, this hypothesis does not explain that co-regulated HTA2 and HTB2 are both seen interacting with ORC2 and ORC6, whereas the differently co-regulated HTA1 and HTB1 do not [44]. Moran et al. [45] show that the promoter region of HTA2 and HTB2 is regulated by the amount of effective H2A+H2B expression. This mechanism is essential for ensuring a sufficient and balanced amount of histones during the S phase–when DNA replication takes place. An excess of H2A+H2B induces a 10-fold decrease in RNA production for HTA1 and HTB1. Thus, a possible explanation for not observing interactions between ORC2/ORC6 and HTA1/HTB1 is that under some circumstances–that might be triggered by the TAP methodology (the fusion of the TAP tag to the C-terminus)–the production of subtypes HTA1 is depressed. Moran et al. argue that the same regulation feed-back takes place for HTB1 as well as for all variants of HHT and HHF [45]. Power Graph Analysis helps to analyze high-throughput data by automatically highlighting the important information: in this case the separation of histones proteins into two differentially co-regulated groups, the P-loop domain containing subunits of the ORC complex and the FACT complex.

### Interaction Profiles of Motif Binding Domains

#### Example 3—Power Graph Analysis of a domain-peptide binding network

**Figure 4:**
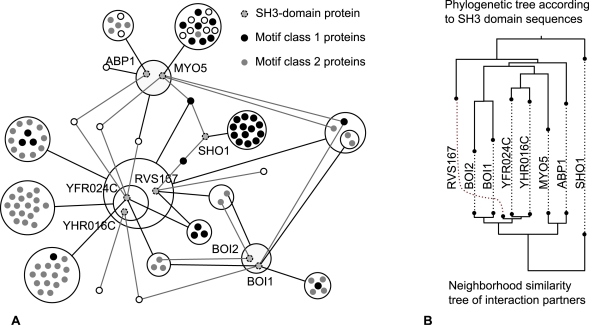
Interactions of SH3 Carrying Proteins. (A) Protein interaction network showing the 105 interaction partners of the SH3 domain carrying proteins: SHO1, ABP1, MYO5, BOI1, BOI2, RVS167, YHR016C and YFR024. The underlying network consists of 182 interactions represented here as 36 power edges–a reduction of 80%–leaving all but only the core information. Class 1 motif (RxxPxxP) proteins are shown in black. Class 2 motif (PxxPxR) proteins are shown in light grey [15]. Note how power graphs group proteins having similar binding motifs together. (B) Phylogeny and interaction profiles. Comparison of the phylogenetic tree of the SH3 domains sequences with the neighbourhood similarity tree of interaction partners. The neighbourhood similarity implied by the power graph reflects the sequence similarity of the SH3 domains.

In reference [15], Landgraf et al. have used a combination of phage display and SPOT synthesis to discover peptides in the yeast proteome that have the potential to bind to eight SH3 domains. Fig. 4A shows a power graph representation of the interaction network of SH3 domain carrying proteins (SHO1, ABP1, MYO5, BOI1, BOI2, RVS167, YHR016C and YFR024). The power graph representation achieves a reduction in complexity by diminishing the number of edges necessary for the representation by 80%. Proteins RVS167, YHR016C and YFR024 are in a power node together showing the similarity of their neighbourhoods. YHR016C and YFR024 are even more similar and have a power node of their own. Proteins that carry the SH3 domain are filled in gray. Power nodes of proteins bound by SH3 carrying proteins are enriched either in motifs of class 1 (RxxPxxP) or in motifs of class 2 (PxxPxR) [15].

#### Domain-interaction profiles correlate to sequence similarity

We investigated how the interaction profiles of these eight SH3 carrying proteins relate to the domain sequences. Fig. 4B shows a strong correlation between the phylogenetic tree of the SH3 domain sequences and the neighbourhood similarity tree of interaction partners. The neighbourhood similarity tree is computed using the proportion of common interaction partners as a similarity measure between two proteins (cf. neighbourhood similarity in methods). As described in the methods section, the hierarchical clustering of nodes according to their neighbourhood similarity is the main principle behind the power graph algorithm.

The pair of SH3-carrying proteins YHR016C/YFR024 that are grouped in one power node in Fig. 4A are also close in the neighbourhood similarity tree. Note how they are also close in the phylogenetic tree. The same holds for the pair BOI1/BOI2. However, we also notice two discrepancies. Proteins ABP1 and MYO5 are grouped together in the neighbourhood similarity tree - whereas they are not in the phylogenetic tree. Protein RVS167 has different placements in the two trees - RVS167 and YHR016C/YFR024 have similar interaction partners but dissimilar sequences.

### Power Graph Analysis Reveals Hidden Structures in Protein Interaction Networks

As we have seen previously on specific examples, power graph analysis can help disentangle complex protein interaction networks. A quantitative analysis requires the definition of measures. Here we introduce the edge reduction measure:
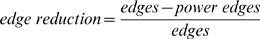
which is the proportion of edges collapsed in the power graph representation. Representing cliques and bicliques with power nodes and power edges allows to trade many edges for a hierarchy of power nodes. Power graphs have less power edges than edges in the original network as these get replaced by power nodes. To take into account the introduction of power nodes, we also compute the removed edge to power node conversion rate:
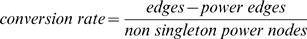



From a visual complexity standpoint, trading edges for a hierarchy of sets of nodes is advantageous since the edges of a clique or biclique necessarily cross in two dimensions, whereas the circles delineating power nodes–by definition–do not.

**Figure 5:**
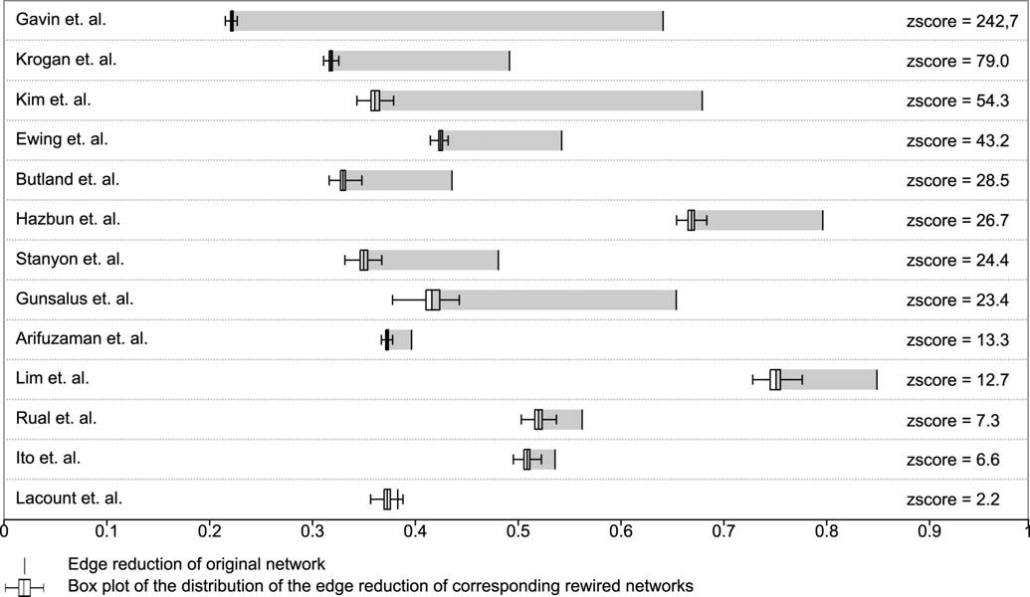
Comparison of 13 Protein Interaction Networks to Corresponding Randomly Rewired Networks. The edge reduction of the rewired networks is represented using a a box-plot. 50% of edge reduction values are inside the box. Most networks exhibit a significant deviation from the null model as indicated by high z-scores between 2.2 and 242.

Table 1 shows the results for 13 protein interaction networks [4], [6], [9], [12], [13], [46]–[53]. The conversion rate is correlated to both the average degree and edge reduction and thus adds little extra information. To evaluate how significant these edge reduction values are, we randomly rewired these networks and then recomputed the corresponding power graphs–thus providing us with a convenient null-model (see methods for random rewiring). Fig. 5 shows the edge reduction for 13 protein interaction networks together with the box-plots for 1000 randomly rewired networks. Computing the power graphs for 1000 rewired networks per protein interaction network allows us to estimate the variance of the edge reduction and thus a z-score. The z-scores obtained indicate that the original networks have significantly higher edge reductions than their rewired counterparts. At one extreme, we have Gavin et al. (2006) with a z-score of 242.

**Table 1:** Power Graph Analysis for 13 Protein Interaction Networks.

Protein Interaction Network	# Nodes	# Edges	Avg. Degree	e.r.	c.r
Lim et al. (2006) [46]	571	701	2.45	85%	12.1
Hazbun et al. (2003) [47]	2243	3130	2.79	79%	13
Kim et al. (2006) [48]	577	1090	3.78	67%	4.1
Gunsalus et al. (2004) [49]	281	514	3.6	65%	4.6
Gavin et al. (2006) [4]	1462	6942	9.4	64%	7.2
Ewing et al. (2007) [50]	2294	6449	5.62	54%	6.6
Ito et al. (2001) [51]	3243	4367	2.69	53%	5.3
Rual et al. (2005) [12]	1527	2529	3.31	50%	4.5
Krogan et al. (2006) [6]	2708	7123	5.26	49%	4.5
Stanyon et al. (2004) [9]	478	1778	7.43	48%	5.3
Stanyon et al. (2004) [9]	478	1778	7.43	48%	5.3
Butland et al. (2005) [52]	1277	5324	8.33	43%	6.0
Arifuzzaman et al. (2006) [53]	2457	8663	7.05	39%	5.4
Lacount et al. (2005) [13]	1272	2643	4.16	38%	3.8

Average degree, edge reduction (e.r.), and edge to power node conversion rate (c.r.).

**Figure 6:**
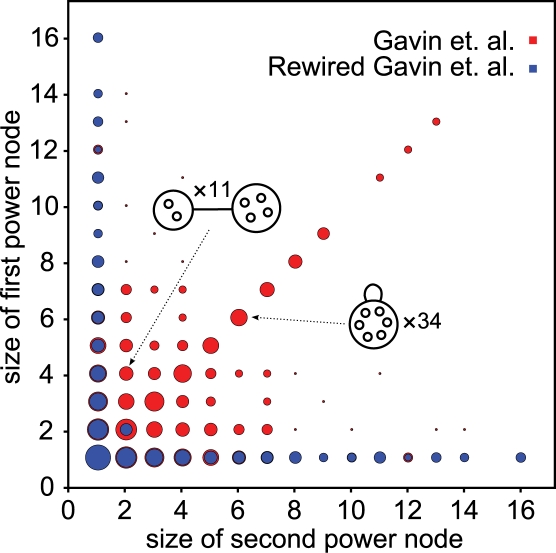
Stars, Bicliques, and Cliques Counts as Obtained through Power Graph Analysis. The area of each disc is proportional to the logarithm of the number of corresponding cliques (diagonal) and bicliques (non-diagonal). Stars are found along the first column or row. For example, there are 11 bicliques between two nodes and 4 nodes, and 34 bicliques of 6 nodes. The diagram is symmetric along the diagonal. Protein interaction networks from Gavin et al. (red) compared to corresponding rewired networks (blue). The high z-score (242) can be explained by significant abundance of cliques and bicliques compared to a random null-model obtained through rewiring. Note that despite the fact that the number of edges is constant, the total count of cliques, bicliques, and stars, is not necessarily constant.

The edge reduction and conversion rate are dependent on the abundance of stars, cliques and bicliques in the network–as these motifs require just one power edge to represent arbitrarily many edges. In particular, from the example previously discussed (casein kinase II complex, nucleosome) we would expect cliques and bicliques to be the culprit. To ascertain that their abundance is indeed the explanation for the higher edge reductions, we examine the count of power edges of different sizes. Fig. 6 shows that power edges representing cliques and bicliques are abundant in the Gavin et al. network, and absent for the corresponding rewired networks. Stars constitute most power edges found in the rewired networks at the exception of bicliques between groups of two nodes. This shows that protein interaction networks have significantly more cliques and bicliques than randomly rewired networks having the same number of nodes, and the same degree distribution.

**Figure 7:**
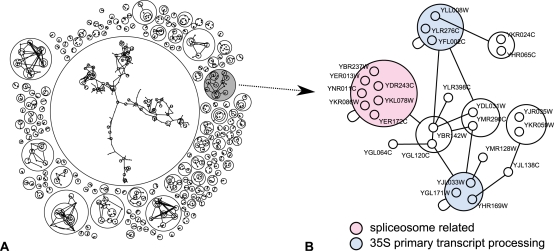
Structural Interaction Network (SIN). (A) Close-up of a *25* node, *68* edges, connected component of the Structural Interaction Network (SIN) [48]. (B) Power graph consisting of 17 power edges, thus an edge reduction of 73%. Three cliques enriched in GO terms related to 35S primary transcript processing and to the spliceosome become explicit in the representation.

Having observed an abundance of cliques and bicliques, there remains the possibility that this is solely caused by experimental or methodological artifacts. However, we know of at least one case for which this cannot be the explanation: the Structural Interaction Network (SIN) by Kim et al. is a set of interactions carefully curated using structural information: all interactions reported are direct physical interactions explained by a known structural binding [48]. This network exhibits a z-score of 54, Fig. 7 shows a close-up of a connected component of the SIN that illustrates its richness in structures: we see three cliques and two bicliques. The three cliques are enriched in Gene Ontology [54] terms related to the spliceosome and to 35S primary transcript processing, thus the proteins of this component are most likely part of the the ribosome and spliceosome machinery. Moreover, it must be said that the examples previously given (casein kinase II complex, nucleosome, domain mediated interactions) in which power graphs give relevant insights on the structure of the networks are often the rule and not an exception. For instance, when analyzed with power graphs, the interaction network of Gavin et al. is–as suggested by the high z-score–very rich in structures that can be related to the known biology.

These results corroborate studies that looked at network motifs identified as functional units in the context of biological networks [55]. Network motifs have been shown to admit generalizations composed of bicliques and stars [56]. These patterns of interaction - characterized by a high connectivity - have been shown to be evolutionary conserved in the yeast protein interaction network [57].

#### Questioning the scale-free hypothesis

It has been argued recently that other distributions than the power-law are a better fit to the observed degree distributions of protein interaction networks [26],[58]. It has also be shown that the scale-free property is not necessarily an intrinsic property of the networks, but could be an artifact caused by selection regularities in the sampling procedures [59],[60]. Other models for protein interaction networks, such as geometric random networks [61] have been shown to be a better fit when looking at the motif composition of protein interaction networks. Our results show that the degree distribution does not characterize completely the idiosyncrasies of protein interaction networks: abundance of stars, cliques and bicliques is an important signature.

### Domain and Gene Ontology Term Enrichment of Power Nodes

To further support the idea that power nodes are not artifacts of the networks topology but have in fact a biological interpretation, we analyzed the enrichment of power nodes in InterPro domains [62],[63] and Gene Ontology (GO) terms [54]. In the previous example on histone proteins, we have an example of a power node of three proteins: ORC1, ORC4, and ORC5, that have in common a P-loop domain.

Our null hypothesis is that “annotations are randomly distributed” following an hyper-geometric distribution. In order to take into account missing domain annotations, only power nodes for which more than two thirds of the proteins are annotated with at least one term or domain are considered. Moreover we use the Bonferroni correction since we do multiple hypothesis testing. Table 2 shows that sufficiently annotated power nodes are significantly enriched in domains, with most p-values below 0.001. Similarly, Table 3 shows the distribution of e-values for the enrichment in GO terms. The p-values for GO terms are not as low as for domains, which would indicate that domains are a better explanation for the occurrence of cliques and bicliques as identified by power graph analysis. Interestingly, when comparing the z-scores found previously and the levels of enrichment both seem to be correlated. For example, the Gavin, Krogan and Kim networks that have the highest z-scores also have the highest overall enrichments of domains and go terms. The Kim et al. network (SIN) has the best overall enrichments for both domains and GO terms, this is in line with the fact that this network is known to be of high quality. Conversely, the power graphs for the Lacount and Lim networks have low z-scores and their power nodes are poorly enriched in InterPro domains or GO terms. These results further confirm the relevance of power graph analysis for analyzing protein interaction networks, in particular the relationship between protein domains and protein interactions.

**Table 2:** Percentage of Power Nodes That Are Significantly Enriched in InterPro Domains.

Network	*p<0.001*	*p<0.01*	n.s.a.
Kim et al. (SIN)(2006) [48]	90%	96%	0%
Krogan et al. (2006) [6]	78%	88%	6%
Gavin et al. (2006) [4]	70%	90%	3%
Rual et al. (2005) [12]	65%	80%	1%
Ewing et al. (2007) [50]	54%	80%	8%
Ito et al. (2001) [51]	51%	86%	7%
Arifuzzaman et al. (2006) [53]	46%	73%	0%
Hazbun et al. (2003) [47]	43%	69%	17%
Butland et al. (2005) [52]	41%	76%	0%
Lim et al. (2006) [46]	39%	56%	10%
Lacount et al. (2005) [13]	20%	54%	29%
Stanyon et al. (2004) [9]	15%	47%	13%

See [62],[63]. Non-sufficiently annotated (n.s.a.) power nodes are not considered (less than two thirds of proteins have annotations). Most power nodes turn out to be enriched at a level of statistical significance of 1 per-thousand. The table is sorted by decreasing overall enrichment.

**Table 3:** Percentage of Power Nodes That Are Significantly Enriched in GO Terms.

Network	*p<0.001*	*p<0.01*	n.s.a.
Kim et al. (SIN)(2006) [48]	63%	89%	0%
Gavin et al. (2006) [4]	58%	73%	0%
Krogan et al. (2006) [6]	51%	60%	1%
Hazbun et al. (2003) [47]	21%	33%	1%
Rual et al. (2005) [12]	19%	35%	1%
Ito et al. (2001) [51]	16%	29%	0%
Ewing et al. (2007) [50]	15%	28%	5%
Butland et al. (2005) [52]	15%	35%	1%
Lim et al. (2006) [46]	11%	29%	0%
Arifuzzaman et al. (2006) [53]	7%	22%	1%
Stanyon et al. (2004) [9]	7%	21%	9%
Lacount et al. (2005) [13]	5%	39%	59%

See [54]. Non-sufficiently annotated (n.s.a.) power nodes are not considered (less than two thirds of proteins have annotations). The table is sorted by decreasing overall enrichment.

### Beyond Protein Interactions

**Figure 8:**
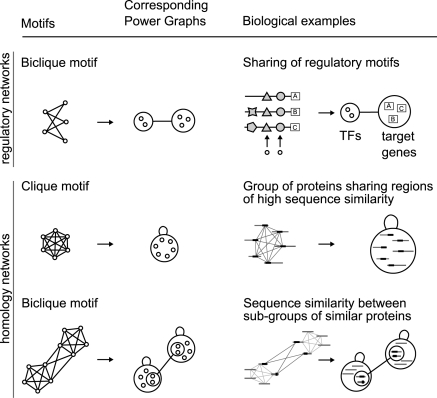
Examples of Occurrences of Bicliques in Gene Regulatory Networks and Homology Networks. Bicliques can occur in regulatory networks due to two reasons: some transcription factors operate within complexes–combinatorial regulation–and regulatory motifs in promoter regions can be shared and repeated for different genes. In the case of homology networks, proteins sharing a sequence region of high similarity–i.e. a domain–induce cliques. Bicliques are similarly induced between sub-groups of similar proteins due additional region of sequence similarity.

Other biological networks benefit from Power Graph Analysis, too. Examples are protein homology networks [16] in which nodes are proteins and edges represent BLAST E-values below a given threshold. These networks are geometric networks defined on the space of sequences with the BLAST E-value as a distance. Geometric networks are known to be saturated in cliques and bicliques [61]. Another example is the analysis of raw gene regulatory networks that also benefits from the Power Graph representation - in particular since gene duplication events tend to create biclique motifs [55],[64]. Fig. 8 illustrates a typical example, in which bicliques arise from the sharing of regulatory motifs. For example, in yeast the genes for histone subunits HTA1 and HTB1 share the same promoter region and are thus under the regulation of the same transcription factors. In the case of homology networks, cliques are often found for groups of highly similar proteins. Bicliques arise between otherwise more distant proteins that share similarity on a specific region i.e. because of a shared domain (Fig. 8). A general principle by which cliques and bicliques occur in biological networks is now apparent: it can be explained by the sharing of sequence regions such as domains, regulatory motifs across different proteins/genes and in general the reuse of building blocks and their subsequent possible combinatorial matchings.

### Example 4—Bipartite Regulatory Networks

**Figure 9:**
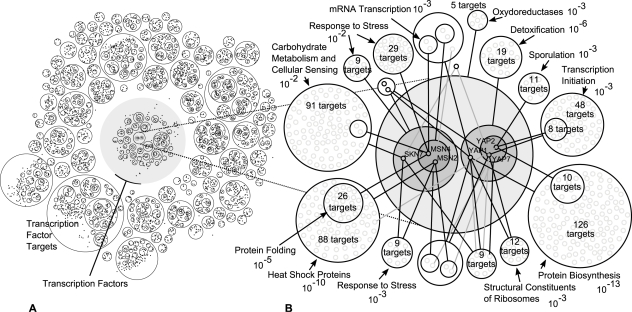
Power Graphs Analysis of a Transcription Regulation Network. (A) Power node hierarchy of the complete bipartite network between *158* transcription factors and 4217 target genes consisting of 13239 assignments between them. (B) Gene targets landscape of a group of transcription factors–SKN7, MSN2, MSN4, YAP1, YAP2(CAD1), and YAP7–regulating the general stress response of S. cerevisiae. Target genes are grouped within power nodes and linked with power edges signifying the assignment of transcription factors to targets. Dominant GO categories in target gene sets are indicated with the order of magnitude of the p-value.

Beyer et al. presented an integrative approach for assigning transcription factors to target genes in *S. cerevisiae* using data from chIP-chip experiments, known binding motifs, clusters of co-expression and other evidences [65]. The result is a probabilistic model with high prediction accuracy, and thus a bipartite network between transcription factors and target genes. The authors identified–among others–YAP1, YAP7 and MSN2 as part of a transcription factor module related to the stress response of S. cerevisiae. To investigate if a similar module could be identified with Power Graph Analysis, we computed the power graph of the whole network and searched the region of the power graph containing YAP1, YAP7 and MSN2. As shown on Fig. 9 a group of transcription factors–SKN7, MSN2, MSN4, YAP1, YAP2(CAD1), and YAP7 are found to have similar gene targets. Two sub groups are identified with differing regulation profiles: SKN7/MSN2/MSN4 and YAP1/YAP2/YAP7. Also shown in Fig. 9, target genes are grouped according to common transcription regulators. For example MSN2 and MSN4 both regulate 26 target genes predominantly involved in protein folding (p-value<10^−5^) and heat shock proteins (p-value<10^−10^). Interestingly, YAP1, YAP2 and YAP7 have in common 19 target genes involved in detoxification (p-value<10^−6^).

The transcription factors MSN2, MSN4, and SKN7 are known to regulate the expression of genes in response to stresses, such as heat and osmotic shock, oxidative stress, low pH, glucose starvation, sorbic acid and high ethanol concentrations [66]. YAP1, YAP2 and YAP7 are similar bZIP proteins of the YAP family characterised by unusual amino acid substitutions of their bZIP domains [67]. It is known that YAP1 and YAP2 are involved in the transcriptional response to drugs, oxidative stress and metal detoxification [66]. YAP7 is however a poorly characterised transcription factor most similar–within the YAP family–to YAP6 whose over expression increases sodium and lithium tolerance [68]. The strong overlap of gene targets of YAP1, YAP2, and YAP7 and the common metal detoxification function of YAP1/YAP2 and YAP6, suggests that YAP7 also plays a role in metal detoxification.

Power Graph Analysis is useful for its ability to decompose a bipartite network into an union of bicliques. This decomposition leads naturally to a hierarchy of clusters of transcription factors linked to a hierarchy of clusters of target genes.

### Example 5—Human Protein Tyrosine Phosphatase Homology Network

The protein tyrosine phosphatase (PTP) family [69] has a central role in signal transduction by controlling the phosphorylation state of tyrosine residues. Tyrosine-specific protein phosphatases (EC:3.1.3.48) catalyse the removal of a phosphate group attached to a tyrosine residue.

**Figure 10:**
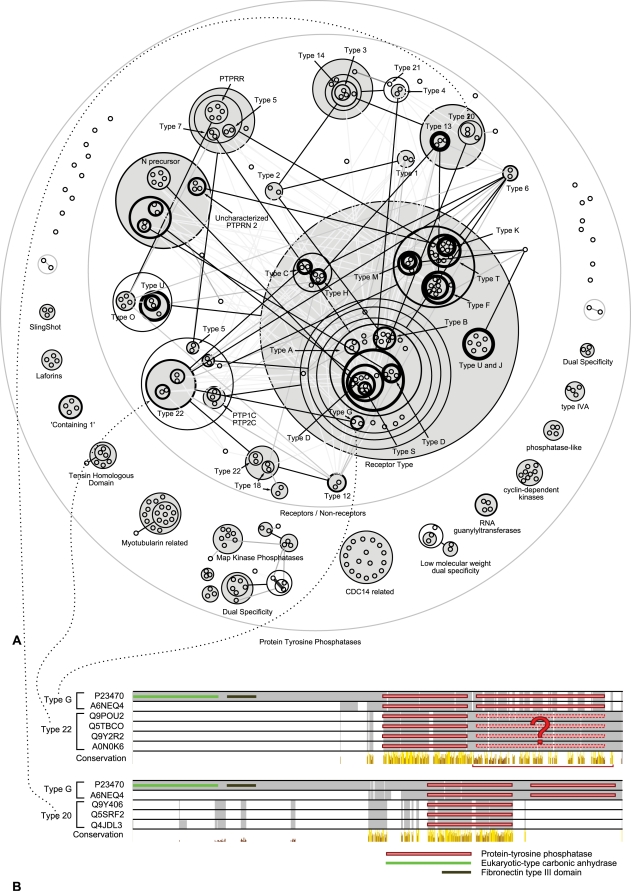
Power Graph Analysis of the Human Protein Tyrosine Phosphatase Homology Network. (A) The original homology network has 279 nodes and 4849 edges. The power graph has 209 power edges - with the addition of 95 non-singleton power nodes. Each node represents a human protein tyrosine phosphatase, with an edge between two proteins corresponding to highly significant alignments with E-values of at most 10^−46^. The network is obtained by an all against all BLASTP scan using the NCBI BLASTP tool [90]. Greyed power nodes correspond to totally connected sets of proteins, for example, all receptor type protein tyrosine phosphatases have an alignment E-value of at least 10^−46^. Black power edges represent many edges of low E-values (lower than 10^−46^), light-gray power edges abstract fewer edges and correspond to less significant sequence similarities. (B) Multiple sequence alignment for type G against type 22 and type G against type 20. The similarity observed in the power graph between type G and type 22 is explained by the homology between a region of type 22 non-receptors and the second copy of the tyrosine phosphatase domain of type G receptors. Negative control: type G and type 20 - which are not linked - do not share this similar region.

The power graph of the protein tyrosine phosphatase homology network is shown in Fig. 10A. The network consists of 279 nodes, each one representing a protein. Edges between two proteins correspond to highly significant alignments of the sequences with a BLASTP E-value of at most 10^−46^. PTPs are usually classified into classical specific phosphatases, dual specificity phosphatases, and other minor classes, such as low molecular weight phosphatases and myotubularins. Classical specific phosphatases are further subdivided into receptor type and non-receptor type. Unsurprisingly, because of their sequence similarities, the categories of receptor, non-receptor, and dual-specificity phosphatases are delineated by the power graph representation. For example the receptor type PTPs are grouped in one power node signifying that they all are similar to one another with E-values below 10^−46^, same for different classes of non-receptor type PTPs, and other, such as myotubularins. Interestingly, the different classes of receptor PTPs, such as types A, B, C, D, F, H, T are discriminated solely on the basis of shared similarity to non-receptor PTPs.

The choice of a threshold for the E-value has an impact on the representation. We observe that for the value of 10^−46^ the power graph reveals the most details. In this case, the lossless reduction in complexity achieved by the power graph representation reaches 95% edge reduction–from 4849 edges to 209 with 95 power nodes. The clustering of proteins in the power graph corresponds to the known classification of PTPs: 82% of leaf power nodes (that do not contain power nodes) have all of their proteins belonging to exactly the same sub-family. While the previous results could have been obtained through the hierarchical clustering of the sequences, Power Graph Analysis reveals additional details.

The cross-links between different regions of the hierarchy constitute a new insight with respect to traditional clustering methods. For example, a group of 6 type B receptor PTPs are linked by a power edge to two type 2 non-receptor PTPs. Fig. 10B shows the multiple alignment of the corresponding sequences. While the common PTP domains are aligned for the six sequences, we also observe that the second copy of the tyrosine phosphatase domain of the two type G PTPs align to an un-annotated region of about 370 amino acids with a sequence identity of 14% and a similarity of 39% (BLOSUM 62). This region corresponds with high probability (NorMD = 1.014) to a non-receptor phosphatase domain listed in ProDom–a database of automatically generated clusters of homologous sequence fragments [70]. To verify that this region is responsible for the high similarity (E-value<10^−46^) between the type G receptor PTPs and type 22 non-receptor PTP, we compared the sequences of type G PTPs to a group of proteins to which they are not connected in the power graph: type 20 PTPs. As Fig. 10B shows, there is no region aligning with the second copy of the phosphatase domain. The previous result suggests that the second phosphatase domain of type 22 PTPs got eroded though the accumulation of mutations following a release in selection pressure.

The detection of similarity cross-links in the hierarchy is the contribution of Power Graph Analysis to the analysis of homology networks. These cross-links constitute a weak signal in networks and are difficult to detect. In this case the evidence for this domain erosion is carried by only eight similarity links between four and two proteins whereas the original network has 4849 edges. In the power graph representation it is one power edge among only 209.

### Robustness Analysis

**Figure 11:**
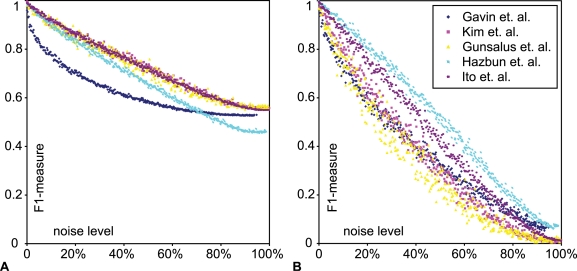
Robustness of Power Graph Analysis through Random Rewiring. Noise level is defined as the number of edges different from the original networks. Random rewiring leaves the total number of edges unchanged, thus a noise level of 100% means that all edges have changed. (A) Comparison of the power node hierarchies. The F1-measure of the precision and recall is computed between the power nodes found for the original network, and power nodes found for the rewired networks. (B) Comparison of the proximity of nodes in the power node hierarchies. Recall is obtained by comparing pairs of nodes together in a power node with the corresponding pairs of nodes in the power graph after random rewiring, the more distant in the power node hierarchy the lower the recall. Precision is obtained by starting from pairs of nodes together in power nodes found in the rewired networks and looking how far–in the power node hierarchy–are the corresponding nodes in the original network. The F1-measure of precision and recall is reported.

Protein networks, and in particular protein interaction networks from high-throughput measurements are known to suffer from many false positives and negatives. To investigate the robustness of power graph analysis, we compare a network's power graph to the power graphs with increasing levels of noise modelled with the addition, removal or rewiring of edges. Fig. 11 shows the results of random rewiring which preserves the degree distribution (see Methods). We used two different evaluation methods and explored the whole range of noise level from 0% to 100%. The first method consists of evaluating the precision and recall of power nodes of power graphs computed on the rewired networks. Note that the F1-measure does not drop to zero at the 100% noise level, this is due to the expectation of random matchings between power nodes which is not zero. The second method focuses on pairs of nodes and aims at evaluating the extent to which nodes remain together in the power node hierarchy after the addition of noise. In both cases, we find that the F1-measure drops proportionally to the level of noise, which shows that power graph analysis is robust to the addition of noise. For some networks such as Gavin et al. the initial losses are higher–characterized by higher tangent slopes around a zero noise level. Whereas other networks such as Ito et al exhibit a stable decrease in the F1-measure. This is in agreement with the previously discussed result in which Gavin et al. was found to contain many cliques and bicliques (high z-score), whereas Ito et al. does not. The high clique and biclique content of Gavin et al. makes it more sensitive to the initial addition of noise. Similar results are obtained for the removal or addition of edges (data not shown).

### Summary and Conclusion

Power Graph Analysis lies at the crossing point of clustering, network motif analysis, information compression, and visualisation. In the previous results, we showed that Power Graph Analysis reveals known underlying biology when applied to protein interaction networks, regulatory and homology networks. It also leads to new insights and new hypotheses. In particular, we presented evidence that the similarity of interaction profiles for peptide-binding SH3 domains correlates with the sequence similarity of these domains. We also discussed how the difference of interaction profiles of otherwise near-identical histone subtypes–visible in the power graph representation–suggests that the TAP methodology interfered with the histone regulatory mechanisms and led to low expression levels of histones subtypes HTA1 and HTB1. Examining other types of networks, we showed that Power Graph Analysis of predicted transcription factors for target genes by Beyer et al. [65] led to the hypothesis that YAP7 is involved in metal detoxification. Finally, Power Graph Analysis, applied to a human phosphatase homology network, reveals similarity cross-links in the hierarchy that are used to spot domain erosion in type 22 non-receptor protein phosphatases.

The main reason behind the usefulness of Power Graph Analysis is the observation that experimental protein interaction networks, bipartite regulatory networks, protein homology networks, and other biological networks have an abundance of cliques and bicliques. Moreover, for small-scale interaction networks and some high quality networks, such as SIN [48] the cliques and bicliques are not solely attributable to noise. The significant enrichment of power nodes in protein domains and Gene Ontology terms further confirms that the cliques and bicliques, that Power Graph Analysis detects, are relevant in the networks. In the case of bipartite regulatory networks, the bipartite nature of the network is ideal for Power Graph Analysis.

Cliques and bicliques in biological networks have been noticed in the past [25]–[27],[71]. Here we argue that this abundance constitutes an important aspect of biological networks in general. Power Graph Analysis distinguishes itself from clustering techniques (socio-affinity clustering [4], RNSC algorithm [17], MCODE algorithm [18], statistical sub-complexes [19]) in that it is specifically designed to identify these cliques and bicliques. Clustering algorithms on graphs often rely on the identification of highly connected regions, abstracting the patterns of connection between groups of nodes. This approach works well for the detection of complexes and other regions of higher connectivity, but it fails for example in the case of the bipartite regulatory networks. In the case of transcriptional regulatory networks, meaningful clusters of transcription factors are not connected to each other but only to target genes. In protein interaction networks, it is also the case that interesting clusters of proteins are defined by their neighbouring proteins and not by their connectivity. For homology networks, we saw that the group of type G receptor PTPs was found because of its similarity to type 22 non-receptor PTPs and not because of a higher level of connectivity.

With Power Graph Analysis it is possible to decompose and represent biological networks as combinations of two simple elements: cliques and bicliques. New analysis methodologies and algorithms can be developed to leverage the information compression made possible by Power Graphs. These directly operate on Power Graphs instead of traditional node-and-edge-graphs. Indeed, one important finding is that the information contained in diverse biological networks, such as protein interaction networks, regulatory networks, and homology networks is highly compressible–even up to 95% for some homology networks. We argue that avoiding this excess of redundant information is possible and desirable.

The advantages and uses of Power Graph Analysis are:

- The simpler representation of complex networks without loss of information.
- Network analysis methodologies and algorithms can be reformulated on top of Power Graph Analysis.
- Cliques and bicliques–which are abundant and relevant for biological networks–are explicitly represented.
- As a side effect of the decomposition, nodes are clustered by connectivity and neighbourhood similarity.
- The connectivity information between these clusters is preserved.

Other graph formalisms have been proposed, such as *hypergraphs* in which hyper-edges are *n-tuples* of nodes [72],[73], or *compound graphs* and *metagraphs* in which nodes are collapsed into *metanodes* [74]. Despite the similarities–such as the collapsing of nodes into metanodes–Power Graphs are different. First, Power Graphs are about decomposing networks using cliques and bicliques. Second, this decomposition is done without loss of information which is usually not the case of compound graphs or metagraphs.

As we showed, Power Graph Analysis is a novel network analysis paradigm that provides a basis for new methodologies. One immediate example is visualisation. Several tools exist to visualise biological networks, such as Cytoscape [75], Pajek [76], Osprey [77], Navigator [78], VisANT [74], ProViz [79], MOVE [80] and GraphViz [81]. However, it is often the case that the amount of information being visualised–the number of edges and edge crossings–makes it difficult to visually explore the networks and mine the desired information. By removing redundant information in the networks, Power Graphs lead to clearer and insightful visualisations. Tools, such as VisANT [74] support the grouping of nodes into clusters which would make the integration of Power Graph Analysis possible. Power graph based visualisation is already available as a plugin for Cytoscape using the described algorithm. Software for computing Power Graphs is available at: http://www.biotec.tu-dresden.de/schroeder/group/powergraphs.

## Methods

### Formal Definition of Power Graphs

Given a graph *G = (V,E)* where *V* is the set of nodes and *E*⊆*V×V* is the set of edges, a *power graph G′ = (V′,E′)* is a graph defined on the power set of nodes *V′*⊆*P(V)* whose elements*–power nodes–*are connected to each other by *power edges*: *E′*⊆*V′×V′*. Hence Power Graphs are defined on the power sets of nodes and power set of edges. The semantics of Power Graphs are as follows: if two power nodes are connected by a power edge in *G′*, this means that in *G* all nodes of the first power node are connected to all nodes of the second power node. Similarly, if a power node is connected to itself by a power edge in *G′*, this signifies that all nodes in the power node are connected to each other by edges in *G*.

The following two conditions are required for simplifying the representations:

- Power node hierarchy condition: Any two power nodes are either disjoint, or one is included in the other.
- Power edge disjointness condition: Each edge of the original graph is represented by one and only one power edge.
- Relaxing the previous two conditions leads to abstract Power Graphs that are difficult to visualize.

### Power Graph Algorithm

We have developed an algorithm for computing near-minimal power graph representations from graphs. The first phase of the algorithm collects candidate power nodes and the second phase uses these to search and add power edges abstracting a maximum number of edges from *G*, which are successively added to the power graph *G′*.

#### First phase: Identifying potential power nodes with hierarchical clustering based on neighbourhood similarity

**Figure 12:**
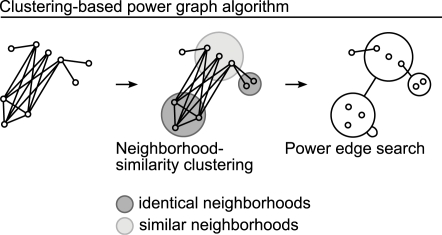
Power graph algorithm. First a neighbourhood similarity clustering of the nodes is performed providing candidate power nodes. In a second step power edges are searched between nodes and candidate power nodes. Note that modular decomposition would not consider as a module the set of nodes having similar but non-identical neighbourhoods. The power graph algorithm finds this candidate and uses it to succinctly represent the biclique.

A set of nodes is a candidate power node if its nodes have neighbours in common. We use a hierarchical clustering algorithm [82] based on neighbourhood similarity to identify such sets. The similarity of two neighbourhoods is the Jaccard index of these two sets [83] (other neighbourhood similarity measures are conceivable). It is always between zero and one: it is zero if the sets *U* and *V* have no common neighbours, and one if both have identical neighbourhoods. Neighbourhood similarity clustering is an intuitive way to identify candidate power nodes. Fig. 12 shows how clustering nodes having identical and similar neighbourhoods provides candidate sets for cliques and bicliques.

To detect stars and other highly asymmetric bicliques in phase two, additional to the hierarchy of sets of nodes achieved with the clustering we add to the candidate power nodes for each node *u* two sets: Its neighbourhood set *N(u)* and the set of common neighbours of the nodes in *N(u)* that contain at least *u*.

#### Second phase: Greedy power edge search

The minimal power graph problem is to be seen as an optimization problem in which the power graph achieving the highest edge reduction is searched. The greedy power edge search follows the heuristic of making the locally optimum decision at each step with the hope of finding the global optimum, or at least a close approximation [84].

Among the candidate power nodes found in phase one each pair that corresponds to a power edge is a candidate power edges. The candidates abstracting the most edges are added successively to the power graph.

#### Related algorithms

The power graph algorithm shares similarities to existing algorithms, such as modular decomposition [2],[85] and spectral clustering [86].

Modular decomposition identifies modules as sets of nodes having *exactly* the same neighbours and builds a tree representation of modules. Algorithms used for modular decompositions can be used for computing Power Graphs, yet they do not achieve as much edge reduction since only modules with strictly identical neighbourhoods are found. For example in Fig. 12 sets of nodes having similar but not identical neighbourhoods are found by the power graph algorithm and used to represent a biclique of three times three edges in the power graph representation, something that would not be found with modular decomposition. Spectral clustering techniques rely on the spectrum of the network's incidence matrix and detect cliques and bicliques as these produce recognizable signals in the spectrum. Other algorithms aim at finding locally maximal bicliques but do not aim at obtaining a balanced decomposition of the whole network [87].

### Scalability of Power Graph Analysis

**Figure 13:**
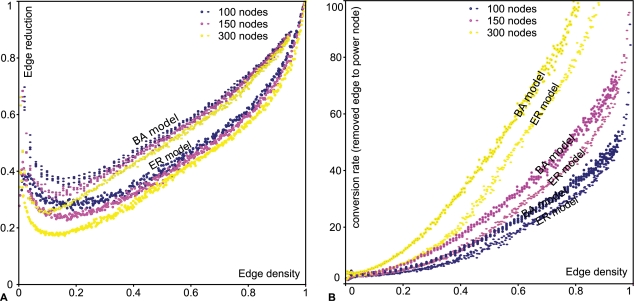
Scalability of Power Graph Analysis. (A) Edge reduction versus edge density. Edge reduction attains a minimum for an edge density between 0.1 and 0.2 and the raises linearly (B) Edge to power node conversion rate versus edge density.

We have conducted experiments to understand the behaviour of the edge reduction for two important classes of networks: synthetic random networks generated according to the Erdös-Rényi model [88] (ER model) and synthetic scale-free networks generated according to the preferential-attachment model of Barabási and Albert (BA model) [24]. Fig. 13 shows how the edge reduction and conversion rate behave for the full range of edge densities. The edge density is the number of edges in the network divided by the maximum number of edges (*n(n−1)/2* where *n* is the number of nodes in the network). For the same edge density, networks generated according to the BA-model are in general more compressible than networks generated using the ER-model. For low edge densities the edge reduction is anti-correlated, it reaches a minimum for an edge density between 0 and 0.2 and then steadily increases toward an edge reduction of 1 for near-clique graphs of edge density close to 1. Increasing the number of edges seems to reduce the border regions (edge density close to 0 or 1) and shifts the curves down to lower edge reductions.

### Random Network Rewiring

Network rewiring is done by choosing randomly two edges *(u,v)* and *(w,t)* and rewiring these to *(u,t)* and *(w,v)*, taking care that these two new edges are not already present in the network. This rewiring step can be repeated a number of times proportional to the number of edges (in our case we chose 16 times). This preserves the degree distribution but removes all correlations between nodes, and thus allows the construction of a null-model for a given network [89].

### Hypergeometric Test

We evaluate the enrichment of a cluster's proteins with domains using p-values assuming an hyper-geometric distribution [17]. The p-value for a cluster of size *C* containing *k*≤*C* proteins with domain *X* is:
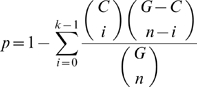



This is the probability that the cluster has *k* or more proteins with domain or GO term *X*, if the cluster's contents were drawn randomly from the set of known proteins. Where *G* is the size of the set of known proteins among which *n*≤*G* have domain *X*. To further take into account the fact that we do multiple tests, we use Bonferroni's correction and compute a corrected p-value *p_c_ = np*, where *n* is the number of annotations tested for a power node.

### Gene and Protein Database Identifiers

The biological function and complex assignments for the examples where obtained through SGD [44] online database. Table 4 recapitulates the names, description and database identifiers of the proteins mentioned in the text.

**Table 4:** Gene and Protein Database Identifiers Mentioned in the Text.

Name	Description	Database ID
CKA1	Alpha catalytic subunit of casein kinase 2	[SGD:YIL035C]
CKA2	Alpha' catalytic subunit of casein kinase 2	[SGD:YOR061W]
CKB1	Beta regulatory subunit of casein kinase 2	[SGD:YGL019W]
CKB2	Beta' catalytic subunit of casein kinase 2	[SGD:YOR039W]
NIP1	Subcomplex (Prt1p-Rpg1p-Nip1p) of eIF3	[SGD:YMR309C]
RPG1	Subcomplex (Prt1p-Rpg1p-Nip1p) of eIF3	[SGD:YBR079C]
PRT1	Sbcomplex (Prt1p-Rpg1p-Nip1p) of eIF3	[SGD:YOR361C]
UTP22	Possible U3 snoRNP protein	[SGD:YGR090W]
ROK1	ATP-dependent RNA helicase of the DEAD box family	[SGD:YGL171W]
RRP7	Involved in rRNA processing and ribosome biogenesis	[SGD:YCL031C]
YLR003C	Uncharacterized, may participate in DNA replication	[SGD:YLR003C]
YKL088W	Predicted phosphopantothenoylcysteine decarboxylase	[SGD:YKL088W]
POB3	Subunit of the FACT complex (RNA Pol II trans. elong.)	[SGD:YML069W]
SPT16	Subunit of the FACT complex (RNA Pol II trans. elong.)	[SGD:YGL207W]
HHO1	Histone H1	[SGD:YPL127C]
HTA1	One of two nearly identical histone H2A subtypes	[SGD:YDR225W]
HTA2	One of two nearly identical histone H2A subtypes	[SGD:YBL003C]
HTB1	One of two nearly identical histone H2B subtypes	[SGD:YDR224C]
HTB2	One of two nearly identical histone H2B subtypes	[SGD:YBL002W]
HHT1	One of two identical histone H3 proteins	[SGD:YBR010W]
HHT2	One of two identical histone H3 proteins	[SGD:YNL031C]
HHF1	One of two identical histone H4 proteins	[SGD:YBR009C]
HHF2	One of two identical histone H4 proteins	[SGD:YNL030W]
HTZ1	Histone variant H2AZ of histone H2A in nucleosomes	[SGD:YOL012C]
ORC1	ORC complex subunit 1, binds on replication origins	[SGD:YML065W]
ORC2	ORC complex subunit 2, binds on replication origins	[SGD:YBR060C]
ORC3	ORC complex subunit 3, binds on replication origins	[SGD:YLL004W]
ORC4	ORC complex subunit 3, binds on replication origins	[SGD:YPR162C]
ORC5	ORC complex subunit 3, binds on replication origins	[SGD:YNL261W]
ORC6	ORC complex subunit 3, binds on replication origins	[SGD:YHR118C]
RVB1	Essential protein involved in transcription vregulation	[SGD:YDR190C]
RVB2	Essential protein involved in transcription bregulation	[SGD:YPL235W]
ARP4	Nuclear actin-related involved in chromatin remodeling	[SGD:YJL081C]
ARP5	Nuclear actin-related involved in chromatin remodeling	[SGD:YNL059C]
SWR1	Swi2/Snf2-related ATPase, SWR1 complex	[SGD:YDR334W]
SWC6	Nucleosome-binding component of the SWR1 complex	[SGD:YML041C]
PIL1	Primary component of eisosomes	[SGD:YGR086C]
SHO1	Transmembrane osmosensor	[SGD:YER118C]
ABP1	Actin-binding protein, cortical actin cytoskeleton	[SGD:YCR088W]
MYO5	One of two type I myosins	[SGD:YMR109W]
BOI1	Polar growth related, functionally redundant with Boi2	[SGD:YBL085W]
BOI2	Polar growth related, functionally redundant with Boi1	[SGD:YGL171W]
RVS167	Actin-associated protein	[SGD:YGL171W]
YSC84	Actin cytoskeleton organization related.	[SGD:Yhr016c]
LSB3	ATP-dependent RNA helicase of the DEAD box family	[SGD:YFR024C-A]
YAP1	bZIP T.F, mediates resistance to cadmium	[SGD:YML007W]
YAP2	AP-1-like bZIP, involved in stress responses	[SGD:YDR423C]
YAP6	Putative bZIP T.F, sodium and lithium tolerance	[SGD:YDR259C]
YAP7	Putative bZIP T.F	[SGD:YOL028C]
MSN2	Transcriptional activator, response to stress	[SGD:YMR037C]
MSN4	Transcriptional activator, response to stress	[SGD:YKL062W]
SKN7	Nuclear response regulator,response to oxidative stress	[SGD:YHR206W]
P23470	Protein-tyrosine phosphatase gamma	[SP:P23470]
A6NEQ4	Uncharacterized Protein-tyrosine phosphatase gamma	[SP:A6NEQ4]
Q9P0U2	Protein tyrosine phosphatase, non-receptor type 22	[SP:Q9P0U2]
Q5TBC0	Protein tyrosine phosphatase, non-receptor type 22	[SP:Q5TBC0]
Q9Y2R2	Tyrosine-protein phosphatase non-receptor type 22	[SP:Q9Y2R2]
A0N0K6	Protein tyrosine phosphatase, non-receptor type 22	[SP:A0N0K6]
Q9Y406	Protein tyrosine phosphatase, non-receptor type 20	[SP:Q9Y406]
Q5SRF2	Protein tyrosine phosphatase, non-receptor type 20	[SP:Q5SRF2]
Q4JDL3	Tyrosine-protein phosphatase non-receptor type 20	[SP:Q4JDL3]
